# Clinical Utility of SNP Array Analysis in Prenatal Diagnosis: A Cohort Study of 5000 Pregnancies

**DOI:** 10.3389/fgene.2020.571219

**Published:** 2020-11-06

**Authors:** Jingjing Xiang, Yang Ding, Xiaoyan Song, Jun Mao, Minjuan Liu, Yinghua Liu, Chao Huang, Qin Zhang, Ting Wang

**Affiliations:** ^1^Center for Reproduction and Genetics, The Affiliated Suzhou Hospital of Nanjing Medical University, Suzhou, China; ^2^Center for Reproduction and Genetics, Suzhou Municipal Hospital, Suzhou, China

**Keywords:** prenatal diagnosis, chromosomal microarray analysis, SNP-array, chromosomal abnormality, copy number variations

## Abstract

**Background:**

Single nucleotide polymorphism array (SNP-array) has been introduced for prenatal diagnosis. We aimed to evaluate the clinical value of SNP-array in the diagnosis of fetal chromosomal anomalies.

**Methods:**

A retrospective study was conducted on 5000 cases tested by SNP-array, and the results of 4022 cases analyzed by both karyotyping and SNP-array were compared.

**Results:**

SNP-array analysis of 5000 samples revealed that the overall abnormality detection rate by SNP-array was 12.3%, and the overall detection rate of clinically significant copy number variations (CNVs) by SNP-array was 2.6%. SNP-array identified clinically significant submicroscopic CNVs in 4.5% fetuses with anomaly on ultrasonography, in 1.6% of fetuses with advanced maternal age (AMA), in 2.5% of fetuses with abnormal result on maternal serum screening, in 2.9% of fetuses with abnormal non-invasive prenatal testing (NIPT) results and in 3.0% of fetuses with other indications. Of the 4022 samples analyzed by both karyotyping and SNP-array, SNP-array could identify all the aneuploidy and triploidy detected by karyotyping but did not identify balanced structural chromosomal abnormalities and low-level mosaicism detected by karyotyping.

**Conclusion:**

SNP-array could additionally identify clinically significant submicroscopic CNVs, and we recommend the combination of SNP-array analysis and karyotyping in prenatal diagnosis.

## Introduction

In the past few years, prenatal diagnosis has expanded from karyotyping and fluorescence *in situ* hybridization (FISH) to chromosomal microarray analysis (CMA). Standard G-banded karyotype analysis is the conventional cytogenetic technique used in prenatal diagnosis, which can detect chromosomal aneuploidies, polyploidies, mosaicism, and structural abnormalities such as balanced or unbalanced translocations or inversions, and deletions or duplications (>5–10 Mb). However, it has several disadvantages such as low resolution, long turnaround time and high rate of culture failure. The development of CMA allows us to identify chromosomal aneuploidy as well as submicroscopic imbalance on the whole genome with short reporting time and higher resolution, and it is recommended as a first-tier approach in prenatal diagnosis for detection of copy number variations (CNVs) in fetuses with structural anomalies observed by ultrasound (American College of Obstetricians and Gynecologists Committee on Genetics, 2013; Society for Maternal-Fetal Medicine et al., 2016). There are two types of CMA: array-based comparative genomic hybridization (aCGH) and single nucleotide polymorphism array (SNP-array). aCGH demonstrates the relative amounts of DNA from various areas of the genome by comparing the test DNA sample with a normal reference DNA sample hence could not detect triploidy, while SNP-array can identify triploidy as well as loss of heterozygosity (LOH) by hybridizing the test sample to the array platform and analyzing the signal intensity of SNP probes (Levy and Wapner, 2018).

Several meta-analyses and systematic reviews have reported that CMA detected clinically significant submicroscopic CNVs in 5.2–10% of fetuses with abnormal fetal ultrasound findings and a normal karyotype (Hillman et al., 2011, 2013; Callaway et al., 2013). However, little is known about the prevalence of clinically significant CNVs in fetuses with structural anomaly in a single anatomical system or with abnormality of ultrasonic soft markers. Furthermore, for pregnant women who chose invasive prenatal diagnosis due to other indications such as advanced maternal age, abnormal result on maternal serum screening and abnormal non-invasive prenatal testing (NIPT) results, there is little information about the diagnostic benefits of SNP-array analysis(Hay et al., 2018).

In this study, we retrospectively reviewed a cohort study of 5000 pregnancies to evaluate the clinical utility of SNP-array analysis in prenatal diagnosis, and explore the improvement of diagnostic yield by CMA over karyotyping. In addition, the distribution of chromosomal abnormalities were also analyzed according to indications for invasive prenatal diagnosis.

## Materials and Methods

### Samples

This study was approved by the institutional Ethics Committee of The Affiliated Suzhou Hospital of Nanjing Medical University. All pregnant women choosing to participate in this study received genetic counseling and provided written informed consent before the invasive prenatal test. A total of 5000 samples including chorionic villi, amniotic fluid and cord blood were collected and analyzed successfully at the center for reproduction and genetics, The affiliated Suzhou hospital of Nanjing medical university, Suzhou, Jiangsu, China from September 2015 through February 2020. Table 1 listed the characteristics and indications for invasive prenatal diagnosis of all the 5000 samples. The mean age of the pregnant women was 32.04 years (range: 18–49 years), and the mean gestational age was 21.28 weeks (range: 9–34 weeks). Parental blood samples were also collected.

**TABLE 1:** The baseline characteristics and indications for prenatal testing of 5000 samples.

	Anomaly on	Advanced maternal	Abnormal result on	Abnormal NIPT		
Characteristics	ultrasonography	age	maternal serum screening	NIPT results	Other^a^	All
Number^b^	1055	1784	1199	515	709	5000
Maternal age (years)^c^	29.48 ± 4.18	37.65 ± 2.29	29.03 ± 3.57	31.38 ± 5.09	29.19 ± 3.04	32.04 ± 5.17
Gestational age (weeks)^c^	23.42 ± 3.40	20.74 ± 1.68	20.90 ± 1.30	21.04 ± 1.68	20.51 ± 1.38	21.28 ± 2.24
Amniotic fluid (no.)	1003	1774	1199	515	704	4941
Chorionic villi (no.)	39	8	0	0	4	44
Cord blood (no.)	13	2	0	0	1	15

*^*a*^Other indications for prenatal testing include history of adverse pregnancy, parental genetic abnormalities, in vitro fertilization, medication use or toxic exposure during pregnancy, consanguineous marriages and parental anxiety.*

*^*b*^As more than one indication may be associated with each sample, the total number of listed five groups is more than 5000.*

*^*c*^Plus-minus values are means ± SD.*

The received amniotic fluid samples were centrifuged immediately to collect amniocytes, and amniocytes were sent for culture if contaminating blood was visualized. 225 (4.6%) of the 4941 amniotic fluid samples were cultured. Chorionic villi were rinsed by saline solution three times and separated using needles under a dissecting microscope. Then genomic DNA of chorionic villi and amniotic fluid were extracted by QIAamp DNA Mini Kit (Qiagen GmbH, Hilden, Germany), and genomic DNA of cord blood was extracted by QIAamp DNA Blood Mini Kit (Qiagen GmbH, Hilden, Germany). Maternal cell contamination were ruled out for all the 5000 samples by short tandem repeat (STR) profiling using Microreader^TM^ 21 (Direct) ID System (Microread, Suzhou, China), which could amplify 20 STR loci and the amelogenin gender marker simultaneously. In addition, 4022 pregnant women chose to perform SNP-array and G-banded karyotyping simultaneously, and 978 pregnant women chose to perform SNP-array only. G-banded karyotyping was performed for 4022 (80.4%) samples according to the principle of ‘An International System for Human Cytogenetic Nomenclature, ISCN2013’ as described previously(Wang et al., 2016).

This was a selective cohort of pregnant women during the study period of Sep 2015 to Feb 2020 who received SNP-array ± karyotyping only. There were women who had karyotyping only during the study period which were not included. There was no detailed information on the number of patients who had maternal serum screening or NIPT in the cohort.

### SNP-Array Analysis

The SNP-array analysis was conducted on the Affymetrix CytoScan platform (Affymetrix, Santa Clara, CA, USA) according to the manufacturer’s protocol. 250 ng genomic DNA was digested, ligated, PCR amplified, purified, fragmented, labeled and hybridized to the Affymetrix Cytoscan 750K array, which includes 550,000 CNV markers and 200,000 SNP markers. After washing, staining and scanning of arrays, raw data were analyzed by Chromosome Analysis Suite (ChAS) 3.2 (Affymetrix, Santa Clara, CA, USA). CNVs were called at a minimum length of 50 Kb containing at least 20 contiguous markers, interpreted and classified as pathogenic (P), likely pathogenic (LP), variants of uncertain significance (VOUS), likely benign (LB) or benign (B), according to the standards and guidelines released by the American College of Medical Genetics (Kearney et al., 2011). Some databases were used as reference resources, including ClinGen Dosage Sensitivity Map^1^, ClinVar^2^, Database of Genomic Variants (DGV)^3^, DECIPHER^4^, Online Mendelian Inheritance in Man (OMIM)^5^, and literatures in PubMed^6^. Microdeletions less than 200 Kb or microduplications less than 500Kb in size and benign CNVs were not reported unless they contained genes known to be associated with disease. We also reported mosaicism greater than 30% and loss of heterozygosity (LOH) of more than 10 Mb in size. If SNP-array detected a CNV in prenatal samples, the parents may choose to perform SNP-array to identify the origin of the CNV. And if a VOUS CNV unrelated to the clinical features was confirmed to be inherited from an unaffected parent, the classification of this CNV will be changed to be likely benign after precluding special considerations listed by the standards and guidelines released by the American College of Medical Genetics (Kearney et al., 2011).

## Results

**FIGURE 1:**
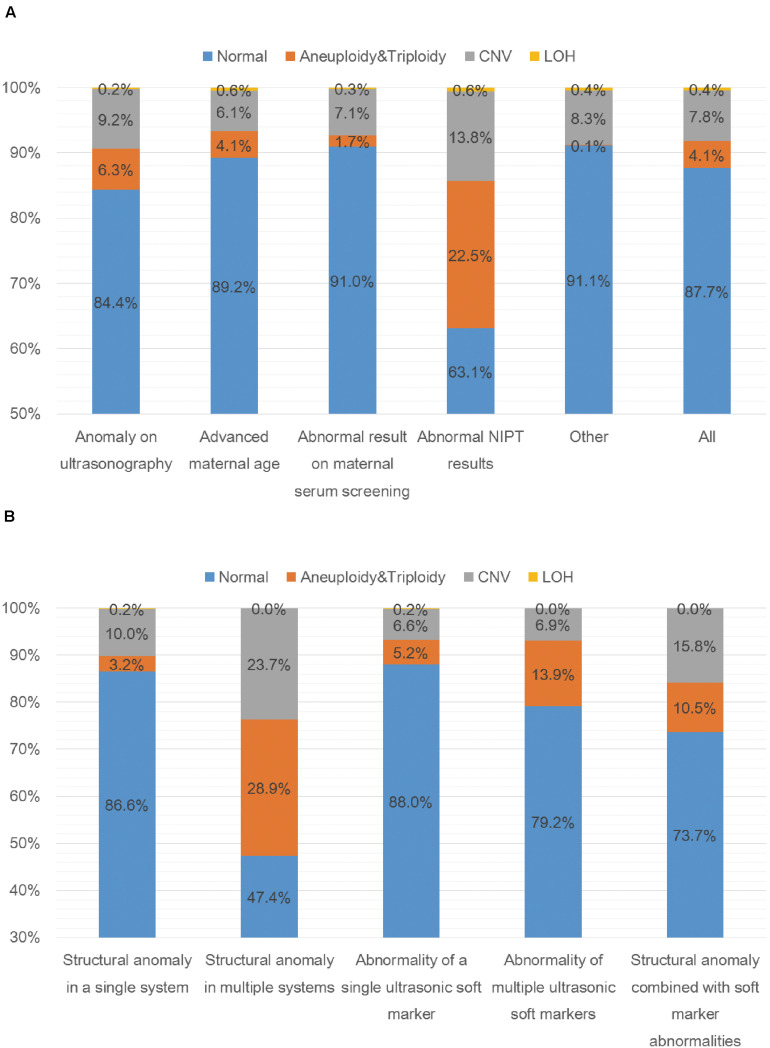
SNP-array results of 5000 samples according to indications for prenatal testing **(A)** and SNP-array results of 1055 samples with anomaly on ultrasonography according to ultrasound characteristics **(B)**. **(A)** The percentage of cases with normal results, aneuploidy & triploidy, CNV and LOH in five subgroups of different indications for prenatal testing and all samples. **(B)** The percentage of cases with normal results, aneuploidy&triploidy, CNV and LOH in five subgroups of different ultrasonic anomalies.

A total of 5000 samples were successfully analyzed by SNP-array from September 2015 to February 2020, and 617 samples of which yielded abnormal results (617/5000, 12.3%), including 207 cases with numerical chromosome anomalies (207/5000, 4.1%), 21 cases with LOH (21/5000, 0.4%) and 389 cases with CNV (389/5000, 7.8%) (Table 2 and Figure 1A). Among 207 cases with numerical chromosome anomalies, trisomy 21(98/5000, 2.0%) was the most common type, followed by sex chromosome aneuploidy (58/5000, 1.2%), trisomy 18 (34/5000, 0.7%) and trisomy 13 (11/5000, 0.2%). Parental samples of 137 cases (137/389, 35.2%) with a total of 144 CNVs (2 CNVs were detected in 5 cases respectively and 3 CNVs were identified in 1 case) were further analyzed by SNP-array, of which 109 CNVs (109/144, 75.7%) were inherited from a parent and 35 CNVs (34/144, 24.3%) were *de novo* in origin. Of 389 cases with CNV, a total of 418 CNVs were identified for 2 CNVs were detected in 27 cases respectively and 3 CNVs were identified in 1 case. One hundred and forty-five CNVs (145/418, 34.7%) were classified as pathogenic (P) or likely pathogenic (LP), 157 CNVs (157/418, 37.6%) were classified as variation of uncertain significance (VOUS), and 116 CNVs (116/418, 27.8%) were classified as likely benign (LB). Moreover, the 5000 samples were categorized into five groups according to the indications for invasive prenatal testing and discussed below (Tables 1, 2 and Figure 1A).

**TABLE 2:** SNP-array results of 5000 samples according to indications for prenatal testing.

Indications	Number^a^	Normal	Aneuploidy& Triploidy^b^	LOH	CNV	CNV^c^			
	
						P	LP	VOUS	LB
Anomaly on ultrasonography	1055	890 (84.4%)	66 (6.3%)	2 (0.2%)	97 (9.2%)	40 (3.8%)	7 (0.7%)	29 (2.7%)	21 (2.0%)
Advanced maternal age (≥35)	1784	1591 (89.2%)	74 (4.1%)	10 (0.6%)	109 (6.1%)	23 (1.3%)	6 (0.3%)	48 (2.7%)	32 (1.8%)
Abnormal result on maternal serum screening	1199	1091 (91.0%)	20 (1.7%)	3 (0.3%)	85 (7.1%)	25 (2.1%)	6 (0.5%)	24 (2.0%)	30 (2.5%)
Abnormal NIPT results	515	325 (63.1%)	116 (22.5%)	3 (0.6%)	71 (13.8%)	9 (1.7%)	6 (1.2%)	37 (7.2%)	19 (3.7%)
Other^d^	709	646 (91.1%)	1 (0.1%)	3 (0.4%)	59 (8.3%)	17 (2.4%)	4 (0.6%)	17 (2.4%)	21 (3.0%)
All	5000	4383 (87.7%)	207 (4.1%)	21 (0.4%)	389 (7.8%)	105 (2.1%)	27 (0.5%)	144 (2.9%)	113 (2.3%)

*^*a*^As more than one indication may be associated with each sample, the total number of listed five categories is more than 5000.*

*^*b*^2 cases of 69,XXX triploidy were identified in the group of anomaly on ultrasonography.*

*^*c*^CNVs were classified into four groups according to interpretation: P, pathogenic; LP, likely pathogenic; VOUS, variation of uncertain significance; LB, likely benign. For 28 cases with more than one CNV, 10 cases with 2 CNVs including a pathogenic CNV and a VOUS CNV were listed in the P group, and the rest 16 cases including 13 cases with 2 P CNVs, 3 cases with 2 VOUS CNVs, 1 case with 2 LB CNVs and 1 case with 3 LB CNVs.*

*^*d*^Other indications for prenatal testing include history of adverse pregnancy, parental genetic abnormalities, *in vitro* fertilization, medication use or toxic exposure during pregnancy, consanguineous marriages and parental anxiety.*

### Anomaly on Ultrasonography

A total of 1055 samples showed fetal anomalies on ultrasound scan, and SNP-array analysis detected 165 samples with chromosome abnormalities (165/1055, 15.6%), including 66 cases with numerical chromosome anomalies (66/1055, 6.3%), 2 cases with LOH (2/1055, 0.2%) and 97 cases with CNV (97/1055, 9.2%) (Table 3 and Figure 1A). The 1055 samples with anomaly on ultrasonography were further divided into five subgroups based on ultrasound characteristics (Edwards and Hui, 2018): 38 cases (38/1055, 3.6%) with structural anomaly in multiple (two or more) systems, 411 cases (411/1055, 39.0%) with structural anomaly in a single system, 458 cases (458/1055, 43.4%) with abnormality of a single ultrasonic soft marker, 72 cases (72/1055, 6.8%) with abnormality of multiple (two or more) ultrasonic soft markers and 76 cases (76/1055, 7.2%) with structural anomaly combined with soft marker abnormalities (Table 3). Overall, the rate of chromosomal abnormalities detected by SNP-array in the group with structural anomaly in multiple systems (20/38, 52.6%) was the highest of the five subgroups, and the rate of chromosomal abnormalities in the group with abnormality of a single ultrasonic soft marker (55/458, 12.0%) was the lowest (Table 3 and Figure 1B). In the subgroup with structural anomaly in a single system, anomaly in cardiovascular system was the most common anomaly (206/411, 50.1%), of which SNP-array detected 14.6% (30/206) chromosomal abnormalities, followed by anomaly in genito-urinary system (62/411, 15.1%) with 11.3% (7/62) chromosomal abnormalities (Table 3). In the subgroup with abnormality of a single ultrasonic soft marker, thickened nuchal translucency (NT) or thickened nuchal fold (NF) was the most common anomaly (173/458, 37.8%) with 13.3% (23/173) chromosomal abnormalities, followed by absent nasal bone (130/458, 28.4%) with 11.5% (15/130) chromosomal abnormalities (Table 3). The prevalence of chromosome aneuploidy/triploidy among cases with three soft markers (mild ventriculomegaly, absent nasal bone, thickened NT or NF) is 6.3% (21/334), while the prevalence of chromosome aneuploidy/triploidy among cases with a single soft marker after exclusion of the above three markers (mild ventriculomegaly, absent NB, thickened NT or NF) is 2.4% (3/124). But the difference was not statistically significant (6.3% vs. 2.4%, chi-squared test, *P* = 0.09881).

**TABLE 3:** SNP-array results of 1055 samples with anomaly on ultrasonography.

Anomalies	Number ^a^	Normal	Aneuploidy &Triploidy ^b^	LOH	CNV	CNV ^c^			
	
						P	LP	VOUS	LB
**Structural anomaly in multiple systems**	**38 (3.6%)**	**18 (47.4%)**	**11 (28.9%)**	**0 (0.00%)**	**9 (23.7%)**	**5 (13.1%)**	**1**	**3**	**0**
**Structural anomaly in a single system**	**411 (39.0%)**	**356 (86.6%)**	**13 (3.2%)**	**1 (0.2%)**	**41 (10.0%)**	**18 (4.4%)**	**0**	**14**	**9**
Central nervous system	13	13	0	0	0	0	0	0	0
Cardiovascular system	206	176 (85.4%)	7 (3.4%)	0 (0.0%)	23 (11.2%)	11 (5.3%)	0	8	4
Gastrointestinal system	37	34 (91.9%)	0 (0.0%)	0 (0.0%)	3 (8.1%)	1 (2.7%)	0	2	0
Respiratory system	11	11	0	0	0	0	0	0	0
Genito-urinary system	62	55 (88.7%)	0 (0.0%)	1 (1.6%)	6 (9.7%)	3 (4.8%)	0	1	2
Musculoskeletal system	42	34 (81.0%)	3 (7.1%)	0 (0.0%)	5 (11.9%)	1 (2.4%)	0	2	2
Faciocervical system	26	22 (84.6%)	3 (11.5%)	0 (0.0%)	1 (3.8%)	0 (0.0%)	0	0	1
Intrauterine growth restriction	14	11	0	0	3	2	0	1	0
**Abnormality of a single ultrasonic soft marker**	**458 (43.4%)**	**403 (88.0%)**	**24 (5.2%)**	**1 (0.2%)**	**30 (6.6%)**	6 (1.3%)	**4**	**10**	**10**
Abnormal amniotic fluid volume	24	23 (95.8%)	0 (0.0%)	0 (0.0%)	1 (4.2%)	1 (4.2%)	0	0	0
Thickened nuchal translucency (NT) or thickened nuchal fold (NF) ^d^	173	150 (86.7%)	14 (8.1%)	0 (0.0%)	9 (5.2%)	3 (1.7%)	1	1	4
Choroid plexus cyst	43	36 (83.7%)	1 (2.3%)	0 (0.0%)	6 (14.0%)	0 (0.0%)	1	3	2
Absent nasal bone	130	115 (88.5%)	7 (5.4%)	1 (0.8%)	7 (5.4%)	1 (0.8%)	2	1	3
Intracardiac echogenic focus	8	8	0	0	0	0	0	0	0
Echogenic intracardiac focus	19	16	0	0	3	0	0	3	0
Mild ventriculomegaly	31	29 (93.5%)	0 (0.0%)	0 (0.0%)	2 (6.5%)	1 (3.2%)	0	1	0
Single umbilical artery	21	18 (85.7%)	1 (4.8%)	0 (0.0%)	2 (9.5%)	0 (0.0%)	0	1	1
Shortened femur	9	8	1	0	0	0	0	0	0
**Abnormality of multiple ultrasonic soft markers**	**72 (6.8%)**	**57 (79.2%)**	**10** (**13.9%**)	**0 (0.0%)**	**5 (6.9%)**	**3 (4.2%)**	**0**	**0**	**2**
**Structural anomaly combined with soft marker abnormalities**	**76 (7.2%)**	**56 (73.7%)**	**8** (**10.5%**)	**0 (0.0%)**	**12 (15.8%)**	**8 (10.5%)**	**2**	**2**	**0**
**Total**	**1055 [100%]**	**890 (84.4%)**	**66** (**6.3%**)	**2 (0.2%)**	**97 (9.2%)**	**40 (3.8%)**	**7**	**29**	**21**

*^*a*^The percentage was not calculated if the total number of the group was less than 20.*

*^*b*^2 cases of 69,XXX triploidy were identified in the group of structural anomaly in multiple systems.*

*^*c*^CNVs were classified into four groups according to interpretation: P, pathogenic; LP, likely pathogenic; VOUS, variation of uncertain significance; LB, likely benign. For cases with 2 CNVs including a pathogenic CNV and a VOUS CNV were listed in the P group.*

*^*d*^Thickened nuchal translucency (NT): NT ≥ 3.0 mm; thickened nuchal fold (NF): NF ≥ 6.0 mm.*

### Advanced Maternal Age (AMA)

**FIGURE 2:**
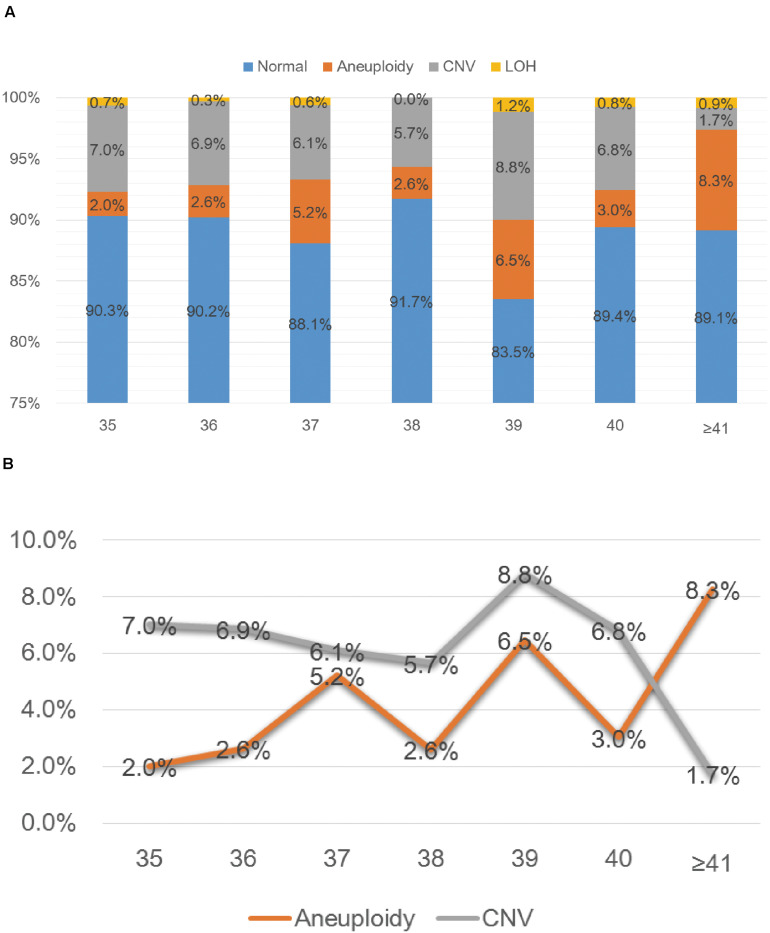
SNP-array results of 1784 samples with advanced maternal age **(A)** and the relationship between age and chromosomal abnormality rate **(B)**. **(A)** The percentage of cases with normal results, aneuploidy, CNV and LOH in seven subgroups of different age. **(B)** The percentage of cases with aneuploidy and CNV according to maternal age.

SNP-array analysis was performed for a total of 1784 pregnant women with AMA (≥ 35 years old), and the average age of this group is 37.65 years (range: 35–49 years) (Table 1). 193 samples were detected with chromosome abnormalities (193/1784, 10.8%), including 74 cases with aneuploidy (74/1784, 4.1%), 10 cases with LOH (10/1784, 0.6%) and 109 cases with CNV (109/1784, 6.1%) (Table 4 and Figure 1A). The 1784 samples with AMA were further divided into seven subgroups according to the maternal age (Table 4 and Figure 2A), and the relationship between maternal age and chromosome abnormality rate was examined. As shown in Figure 2B, the rate of aneuploidy significantly increased with age, while there was no such trend in the rate of CNVs.

**TABLE 4:** SNP-array results of 1784 samples with advanced maternal age (≥35).

Age	Number	Normal	Aneuploidy	LOH	CNV	CNV ^a^			
	
						P	LP	VOUS	LB
35	299 (16.8%)	270 (90.3%)	6 (2.0%)	2 (0.7%)	21 (7.0%)	4(1.3%)	1	10	6
36	379 (21.2%)	342 (90.2%)	10 (2.6%)	1 (0.3%)	26 (6.9%)	7(1.8%)	2	13	4
37	344 (19.3%)	303 (88.1%)	18 (5.2%)	2 (0.6%)	21 (6.1%)	5(1.5%)	0	8	8
38	230 (12.9%)	211 (91.7%)	6 (2.6%)	0 (0.0%)	13 (5.7%)	1(0.4%)	2	6	4
39	170 (9.5%)	142 (83.5%)	11 (6.5%)	2 (1.2%)	15 (8.8%)	3(1.8%)	1	6	5
40	132 (7.4%)	118 (89.4%)	4 (3.0%)	1 (0.8%)	9 (6.8%)	3(2.2%)	0	2	4
≥41	230 (12.9%)	205 (89.1%)	19 (8.3%)	2 (0.9%)	4 (1.7%)	0(0.0%)	0	3	1
Total	1784 [100]	1591 (89.2%)	74 (4.1%)	10 (0.6%)	109 (6.1%)	23(1.3%)	6	48	32

*^*a*^CNVs were classified into four groups according to interpretation: P, pathogenic; LP, likely pathogenic; VOUS, variation of uncertain significance; LB, likely benign. For cases with 2 CNVs including a pathogenic CNV and a VOUS CNV were listed in the P group.*

### Abnormal Result on Maternal Serum Screening

In our center, second trimester maternal serum screening was conducted by measuring AFP and free β-HCG levels in maternal serum. A total of 1199 samples with abnormal result on maternal serum screening were analyzed by SNP-array, and abnormal results were observed in 108 samples (108/1199, 9.0%), including 21 cases with aneuploidy (20/1199, 1.7%), 3 cases with LOH (3/1199, 0.3%) and 85 cases with CNV (85/1199, 7.1%) (Table 5). The 1199 samples with abnormal result on maternal serum screening were divided into four subgroups according to risk type: 998 cases (998/1199, 83.2%) with high risk of trisomy 21 syndrome, 68 cases (68/1199, 5.7%) with high risk of trisomy 18 syndrome, 24 cases (24/1199, 2.0%) with high risk of open neural tube defects (ONTD) and 109 cases (109/1199, 9.1%) with intermediate risk of trisomy 21 syndrome (Table 5). Among these, SNP-array confirmed 11 samples of trisomy 21, including 7 cases in the group of high risk of trisomy 21 syndrome and 4 cases in the group of intermediate risk of trisomy 21 syndrome. And all the identified 4 samples of trisomy 18 showed high risk of trisomy 18 syndrome (Table 5).

**TABLE 5:** SNP-array results of 1199 samples with abnormal result on maternal serum screening.

Abnormality	Number	Normal	Aneuploidy	LOH	CNV	CNV^a^			
	
						P	LP	VOUS	LB
High risk of trisomy 21 syndrome (Down syndrome, DS) (≥1/300)	998 (83.2%)	917 (91.9%)	11 (1.2%)	3 (0.3%)	66 (6.6%)	17(1.7%)	5	22	22
High risk of trisomy 18 syndrome (Edwards syndrome, ES) (≥1/350)	68 (5.7%)	57 (83.8%)	4 (5.9%)	0 (0.0%)	7 (10.3%)	3(4.4%)	1	0	3
High risk of open neural tube defects (ONTD) (AFP ≥ 2.5 MoM)^b^	24 (2.0%)	22 (91.7%)	0 (0.0%)	0 (0.0%)	2 (8.3%)	0(0.0%)	0	1	1
Intermediate risk of trisomy 21 syndrome (Down syndrome, DS) (1/301–1/1000)	109 (9.1%)	95 (87.2%)	4 (3.7%)	0 (0.0%)	10 (9.2%)	5(4.6%)	0	1	4
Total	1199 [100%]	1091 (91.0%)	20 (1.7%)	3 (0.3%)	85 (7.1%)	25(2.1%)	6	24	30

*^*a*^CNVs were classified into four groups according to interpretation: P, pathogenic; LP, likely pathogenic; VOUS, variation of uncertain significance; LB, likely benign. For cases with 2 CNVs including a pathogenic CNV and a VOUS CNV were listed in the P group.*

*^*b*^AFP and freeβ-HCG levels in maternal serum were converted to MoM (multiples of median).*

### Abnormal NIPT Results

Currently, whole-genome sequencing-based NIPT could be used for detecting aneuploidy as well as genome-wide CNVs (> 10 Mb) (Liu et al., 2016). SNP-array analysis was performed to verify the abnormal NIPT results (including chromosome aneuploidies and CNVs detected by NIPT) of 515 samples, and 190 samples (190/515, 36.9%) were identified with chromosome abnormalities, including 116 cases with aneuploidy (116/515, 22.5%), 3 cases with LOH (3/515, 0.6%), and 71 cases with CNV (71/515, 13.8%) (Table 6). The 515 samples with abnormal NIPT results were divided into five subgroups based on chromosome locations of abnormalities. In the group of abnormality in chr21, 51 cases of trisomy 21 and 2 cases of CNVs on chr21 were confirmed by SNP-array. SNP-array analysis validated 7 cases of trisomy 13 and 4 cases of CNVs on chr13 in the group of abnormality in chr13. In the group of abnormality in chr18, SNP-array verified 16 cases of trisomy 18, 1 case of uniparental disomy (UPD) of chr18 and 7 cases of CNVs on chr18. In addition, in the group of abnormality in other autosomes, SNP-array validated 1 case of mosaic trisomy 22, 1 case of UPD of chr8, 1 case of LOH on chr4 and 30 cases of CNVs on autosomes except chr21, chr13 and chr18. And 39 cases of aneuploidy and 4 cases of CNVs on sex chromosomes were confirmed by SNP-array in the group of abnormality in sex chromosomes (Table 6 and Supplementary Table 1).

**TABLE 6:** SNP-array results of 515 samples with abnormal NIPT results.

Abnormality	Number	Normal	Aneuploidy	LOH	CNV	CNV^a^			
	
						P	LP	VOUS	LB
Chr21	105 (20.4%)	48 (45.7%)	52 (49.5%)	0 (0.00%)	5 (4.8%)	1(1.0%)	0	2	2
Chr13	46 (8.9%)	33 (71.7%)	7 (15.2%)	0 (0.00%)	6 (13.0%)	0(0.0%)	0	5	1
Chr18	41 (8.0%)	16 (39.0%)	16 (39.0%)	1 (2.4%)	8 (19.5%)	2(4.9%)	0	3	3
Other autosomes^b^	185 (36.1%)	139 (75.1%)	1 (0.5%)	2 (1.1%)	43 (23.2%)	5(2.7%)	5	24	9
Sex chromosomes	138 (26.6%)	89 (64.5%)	40 (29.0%)	0 (0.00%)	9 (6.5%)	1(0.7%)	1	3	4
Total	515 [100%]	325 (63.1%)	116 (22.5%)	3 (0.6%)	71 (13.8%)	9(1.7%)	6	37	19

*^*a*^CNVs were classified into four groups according to interpretation: P, pathogenic; LP, likely pathogenic; VOUS, variation of uncertain significance; LB, likely benign. For cases with 2 CNVs including a pathogenic CNV and a VOUS CNV were listed in the P group.*

*^*b*^Other autosomes: autosomes except chr21, chr13, and chr18.*

### Other Indications

The rest (709 samples) had other indications for prenatal testing, such as history of adverse pregnancy, parental genetic abnormalities, *in vitro* fertilization, medication use or toxic exposure during pregnancy, consanguineous marriages and parental anxiety (Table 1). The results of SNP-array revealed that 63 samples (63/709, 8.9%) had chromosomal abnormalities, including 1 case with aneuploidy (47,XYY), 3 cases with LOH and 59 cases with CNV (Table 2).

### Discrepancy Between Karyotyping and SNP-Array

Traditional karyotype analysis were performed simultaneously on 4022 samples (4022/5000, 80.4%) of our cohort (Table 7). Of these, 151 cases of aneuploidy and 2 cases of triploidy (69,XXX) were both identified by karyotyping and SNP-array. Among 3665 cases with normal karyotype, SNP-array analysis yielded 286 abnormal results (286/3665, 7.8%) including 2 cases of mosaic 45,X, 19 cases of LOH and 265 cases of microduplication/microdeletion, the size of 278 CNVs (2 CNVs were identified in 11 cases respectively and 3 CNVs were identified in 1 case) ranged from 99 kb to 6.287 Mb. Of 19 mosaic cases identified by karyotyping, 6 cases were not detected by SNP-array including 3 cases of low-level mosaic aneuploidy and 3 cases of mosaic balanced structural rearrangement, while SNP-array additionally detected 1 case of LOH and 1 case of microduplication (Table 7 and Supplementary Figure 1). Among 185 cases with structural rearrangement revealed by karyotyping, 147 cases of structural rearrangement including balanced structural rearrangement, chromosomal heteromorphisms and marker chromosomes were not detected by SNP-array as expected, while SNP-array additionally detected 1 case of mosaic trisomy 22 and 10 cases of microduplication/microdeletion (Table 7).

**TABLE 7:** The results of karyotype and SNP-array Analysis in 4022 samples.

Classification	Detected by Karyotyping (no.)	Consistent with SNP-array results (no.)
Normal	3665	3379^a^
Trisomy 21	75	75
Trisomy 18	29	29
Trisomy 13	9	9
48,XXY, +18	1	1
45,X	5	5
47,XXY	25	25
47,XYY	3	3
47,XXX	4	4
69,XXX	2	2
Mosaic	19	13^b^
Structural rearrangement	185	27^c^
Total	4022	3572

*^*a*^SNP-array analysis revealed additional 286 abnormal results including 2 cases of mosaic 45, X, 19 cases of LOH and 265 cases of microduplication/microdeletion.*

*^*b*^3 cases of low-level mosaic aneuploidy (47,XY, + 20[6]/46,XY[44]; 47,XX, + 18[2]/46,XX[68]; 47,XX, + 2[4]/46,XX[55]) and 3 cases of mosaic balanced structural rearrangement (46, XY,t(5;16)(q15;p10)[3]/46, XY [47]; 46,XX,t(1;3)(p10;q10)[4]/46,XX[46]; 46,X,inv(Y)(p11.2q11.23)[34]/46,X,inv(Y)(p11.2q11.23),t(1;16)(q21;q13.1)[16]) were not detected by SNP-array, while SNP-array additionally detected 1 case of LOH and 1 case of microduplication (Supplementary Figure 1).*

*^*c*^147 cases of structural rearrangement including balanced structural rearrangement, chromosomal heteromorphisms and marker chromosomes were not detected by SNP-array, while SNP-array additionally detected 1 case of mosaic trisomy 22 and 10 cases of microduplication/microdeletion.*

## Discussion

In this study, we investigated the clinical value of SNP-array in prenatal diagnosis in a cohort of 5000 pregnancies. The detection rate of abnormalities by SNP-array was 12.3%, including 4.1% of cases with numerical chromosome anomalies, 0.4% with LOH and 7.8% with CNV (Table 2 and Figure 1A). And the detection rate of clinically significant CNVs (i.e., CNVs classified as P or LP) by SNP-array was 2.6% (Table 2), which is similar to a previous systematic review that overall 295/12362 (2.4%) have CNVs with associated clinical significance (Callaway et al., 2013) and a recent retrospective analysis of 10-year data reported that the abnormality detection rate of pathogenic CNVs by aCGH is 2.59% for prenatal cases (Chai et al., 2019). Moreover, the 5000 samples were categorized into five groups according to the indications for invasive prenatal testing: anomaly on ultrasonography, advanced maternal age, abnormal result on maternal serum screening, abnormal NIPT results and other indications. The rate of chromosomal abnormalities detected by SNP-array in the group with abnormal NIPT results was the highest (190/515, 36.9%), followed by the group with anomaly on ultrasonography (165/1055, 15.6%), and the rate of chromosomal abnormalities in the group with other indications was the lowest (63/709, 8.9%) (Table 2 and Figure 1A).

In the group of anomaly on ultrasonography, SNP-array identified 4.5% (47/1055) clinically significant submicroscopic CNVs in fetuses with abnormal prenatal ultrasound findings (Table 2), which is lower than previous meta-analyses that CMA identified 5.2%–10% additional chromosome aberrations over conventional karyotyping in fetuses with a structural malformation on ultrasound (Hillman et al., 2011, 2013; Callaway et al., 2013). This might result from the addition of samples with ultrasonic soft marker abnormalities into this group. A systematic review revealed that a causative submicroscopic CNV could be identified in 3.1–7.9% of fetuses with ultrasound anomaly restricted to one system and in 9.1% of fetuses with multiple ultrasound anomalies (de Wit et al., 2014). In our study, the fetuses with structural anomaly in a single system had a 4.4% (18/411) chance of carrying clinically significant CNV, and the chance increased to 15.8% (6/38) for fetuses with structural anomaly in multiple systems and to 13.2% (10/76) for fetuses with structural anomaly combined with soft marker abnormalities (Table 3). In the subgroup of structural anomaly in a single system, clinically significant CNVs most commonly associated with cardiovascular (11/206, 5.3%) and genito-urinary systems (3/62, 4.8%) (Table 3), which is in accordance with a previous report that the renal and cardiac systems were significantly associated with other-than-common benign CNVs (Donnelly et al., 2014). The detection rates of clinically significant submicroscopic CNVs in fetuses with abnormality of a single ultrasonic soft marker and multiple ultrasonic soft markers were 2.2% (10/458) and 4.2% (3/72) respectively (Table 3), which is consistent with a previous report that clinically significant genomic alterations were identified in 2.6% of cases with anomalies of ultrasonic soft markers (Shaffer et al., 2012). Recently, a research revealed a small but statistically insignificant increase in odds of clinically relevant CNV in fetuses/children with one or more ultrasound soft markers, which is in consistency with our result that no statistically significant difference was noted between rates of clinically significant submicroscopic CNVs in fetuses with abnormality of a single ultrasonic soft marker and multiple ultrasonic soft markers (2.2% vs. 4.2%, chi-squared test, *P* = 0.55) (Angras et al., 2020).

In the group of AMA, clinically significant submicroscopic CNVs were detected by SNP-array in 1.6% (29/1784) of fetuses with AMA (Table 2), which is similar to the result of a previous multicenter study by the National Institute of Child Health and Human Development (NICHD) that a submicroscopic pathogenic aberration was detected prenatally in 1.7% (95% CI 1.2–2.4) of fetuses tested due to AMA (Wapner et al., 2012). And our results demonstrated that the rate of numerical chromosome anomalies significantly increased with age, while there was no such trend in the rate of CNVs for fetuses with AMA, which is consistent with previous studies (Grati et al., 2015; Shi et al., 2019).

In the group of abnormal result on maternal serum screening, SNP-array identified clinically significant submicroscopic CNVs in 2.5% (30/1199) of fetuses with abnormal result on maternal serum screening (Table 2), which is consistent with the result of a previous NICHD multicenter study that 1.6% (95% CI: 0.9–2.9) of fetuses tested abnormal on Down’s syndrome screening had clinically relevant findings on microarray that were not detected on karyotyping (Wapner et al., 2012).

In the group of abnormal NIPT results, SNP-array analysis detected clinically significant submicroscopic CNVs in 2.9% (15/515) of fetuses with abnormal NIPT results, while in the group of other indications, clinically significant submicroscopic CNVs were detected by SNP-array in 3.0% (21/709) of fetuses with other indications (Table 2). In this study, abnormal NIPT results included chromosome aneuploidies and CNVs of all 24 chromosomes detected by NIPT. NIPT has been widely applied for detecting fetal chromosome trisomy 13, 18, and 21 (T13, T18, and T21) and sex chromosome aneuploidies. However, the performance of NIPT for screening other fetal chromosome aneuploidies and CNVs is still limited. In 2019, we have evaluated the clinical practical performance of NIPT to analyze all 24 chromosome aneuploidies among 57,204 pregnancies in our center, and concluded that NIPT showed good performance for detecting T13, T18, and T21, but the accuracy rate of NIPT for detecting rare fetal chromosome aneuploidies was insufficient (Xue et al., 2019). In this study, only cases with abnormal NIPT results were analyzed by SNP-array, while the SNP-array or karyotyping results of cases with normal NIPT results were unknown, and the abnormal NIPT results of 516 cases were given by different NIPT platforms in our center or even other centers. Therefore it is difficult to calculate the false positive rate of NIPT in this study.

We also compare the results of karyotype and SNP-array analysis on 4022 samples (4022/5000, 80.4%) of our cohort. As SNP-array can identify submicroscopic CNVs and LOH not detected on karyotyping, 286 abnormal results (286/4022, 7.1%) were additionally revealed by SNP-array analysis over G-banded karyotyping. Apart from 2 cases of mosaic 45, X, SNP-array analysis detected clinically significant submicroscopic CNVs in 2.9% (64/3665, 1.7%) of fetuses with a normal karyotype. And the discrepant results between karyotyping and SNP-array analysis for samples with mosaicism or structural rearrangement detected by karyotyping could be due to direct (uncultured) analysis by SNP-array, and structural rearrangement (including balanced structural rearrangement, chromosomal heteromorphisms and marker chromosomes) and low-level mosaicism (<10–15%) not detectable by SNP-array. As conventional karyotyping is still valuable in the identification of balanced structural chromosomal abnormalities and low-level mosaicism, we recommend the combined application of karyotyping and SNP-array analysis in prenatal diagnosis.

In this cohort, the detection rate of VOUS CNVs by SNP-array was 2.9% (Table 2), which is higher than that of a previous NICHD multicenter study (1.6%) (Wapner et al., 2012) and lower than that of another cohort of 5026 pregnancies (4.6%) (Wang et al., 2019). These differences may result from different reporting criteria and interpretation deviations. Moreover, reporting VOUS and LB CNVs may increase patient anxiety and the genetic counseling workload in clinical practice.

In summary, a retrospective analysis was performed on a cohort of 5000 pregnancies, and the detection rate of chromosome abnormalities by SNP-array was 12.3%, including 4.1% of cases with numerical chromosome anomalies, 0.4% with LOH and 7.8% with CNVs. The overall detection rate of clinically significant CNVs by SNP-array was 2.6%, and we recommend the combined application of karyotyping and SNP-array analysis in prenatal diagnosis. According to the indications for invasive prenatal testing, SNP-array identified clinically significant submicroscopic CNVs in 4.5% fetuses with anomaly on ultrasonography, in 1.6% of fetuses with AMA, in 2.5% of fetuses with abnormal result on maternal serum screening, in 2.9% of fetuses with abnormal NIPT results and in 3.0% of fetuses with other indications.

## Data Availability Statement

The genotyping data for this article are not publicly available to assure patient confidentiality and participant privacy. Requests to access the datasets should be directed to TW, biowt@njmu.edu.cn.

## Ethics Statement

The studies involving human participants were reviewed and approved by the institutional Ethics Committee of The Affiliated Suzhou Hospital of Nanjing Medical University. Written informed consent to participate in this study was provided by the participants’ legal guardian/next of kin.

## Author Contributions

JX, QZ, and TW were responsible for testing strategy design and manuscript preparation. XS and CH carried out the SNP-array analysis. JX, JM, ML, and YL performed data analysis and interpretation. YD and QZ conducted genetic counseling. All authors read and approved the final manuscript.

## Conflict of Interest

The authors declare that the research was conducted in the absence of any commercial or financial relationships that could be construed as a potential conflict of interest.

## Footnotes

1 https://www.ncbi.nlm.nih.gov/projects/dbvar/clingen

2 https://www.ncbi.nlm.nih.gov/clinvar/

3 http://projects.tcag.ca/variation

4 http://decipher.sanger.ac.uk/

5 http://www.omim.org

6 https://pubmed.ncbi.nlm.nih.gov/

## Supplementary Material

Supplementary Figure 1 The SNP-array results of four cases with low-level mosaic aneuploidy detected by karyotyping. Left panel show the weighted Log2 ratio plot of all chromosomes and the LOH and BAF plot of abnormal chromosomes respectively, and right panel show the karyotyping results. **(A)** For the case of 45,X[7]/46,XX[43], the mosaic level of 45,X was 14% that could be observed in the weighted Log2 ratio plot and the BAF plot of chromosome X, and SNP-array additionally detected a LB CNV on chromosome 16 (arr[hg19] 16q23.1(77,162,977–79,080,183)x3,1.917 Mb, maternal origin); **(B)** For the case of 47,XY, + 20[6]/46,XY[44], the mosaic level of 47,XY, + 20 was 12% that could not be observed in the weighted Log2 ratio plot and the BAF plot of chromosome 20, and SNP-array additionally detected two large segments of LOH on chromosome 20 (arr[hg19] 20q11.21q13.2(29,510,306–52,406,127) hmz, 22.895Mb, maternal origin;arr[hg19] 20p12.1p11.1(16,873,264–26,266,313) hmz, 9.393 Mb, maternal origin); **(C)** For the case of 47,XX, + 18[2]/46,XX[68]; the mosaic level of 47,XX, + 18 was 2.9% that could not be observed in the weighted Log2 ratio plot and the BAF plot of chromosome 18, and the SNP-array result is normal; **(D)** For the case of 47,XX, + 2[4]/46,XX[55], the mosaic level of 47,XX, + 2 was 6.8% that could not be observed in the weighted Log2 ratio plot, and the BAF plot of chromosome 2 could give some hint on mosaicism, while the SNP-array result is normal for mosaicism level less than 30% was not reported.
