# Supplementary figures and images for: Clinical Utility of SNP Array Analysis in Prenatal Diagnosis: A Cohort Study of 5000 Pregnancies
Source: Front Genet. 2020 Nov 6;11:571219. doi: 10.3389/fgene.2020.571219 (PMC7677511; doi:10.3389/fgene.2020.571219)

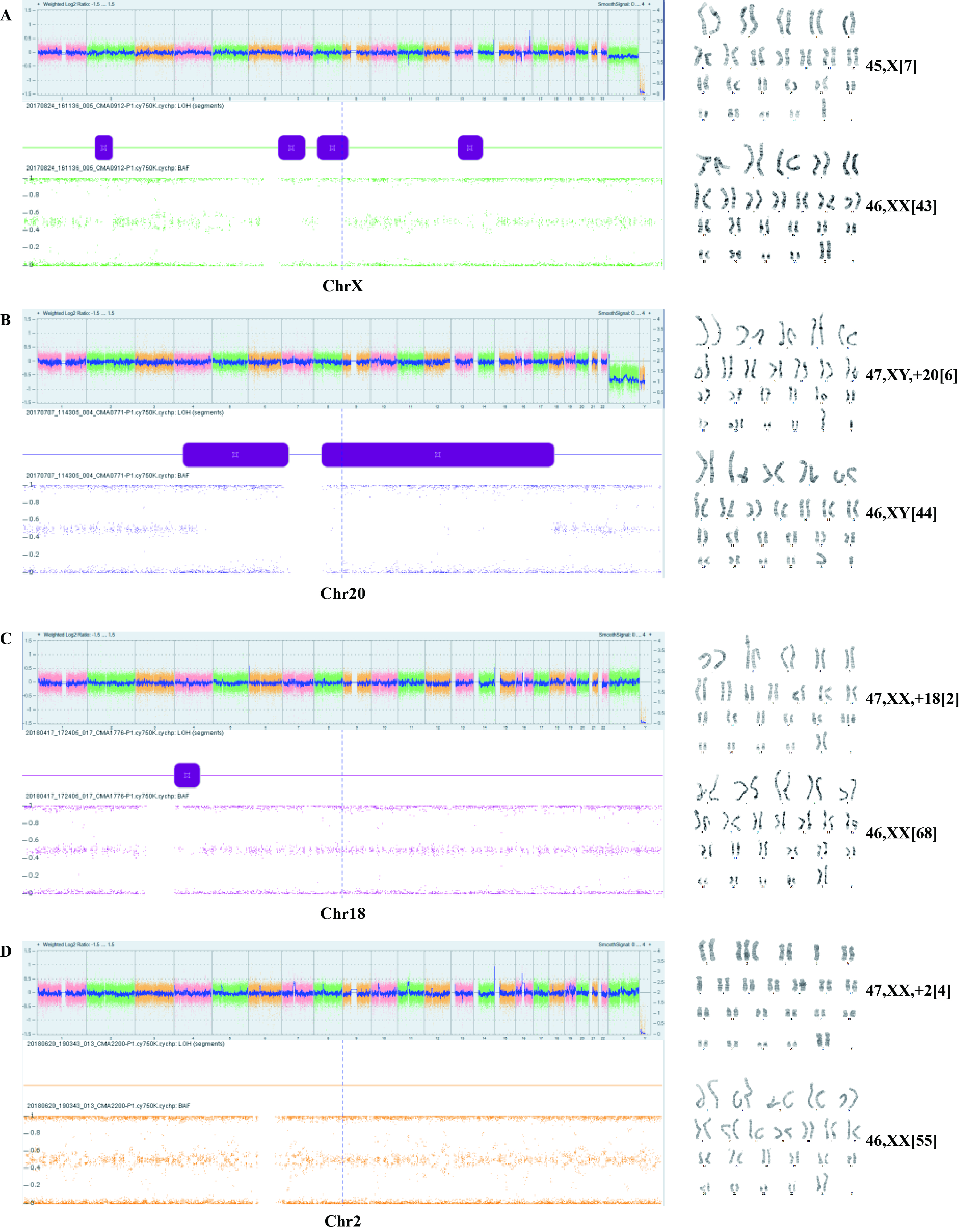

Supplement: Supplementary Figure 1 — The SNP-array results of four cases with low-level mosaic aneuploidy detected by karyotyping. Left panel show the weighted Log2 ratio plot of all chromosomes and the LOH and BAF plot of abnormal chromosomes respectively, and right panel show the karyotyping results. (A) For the case of 45,X[7]/46,XX[43], the mosaic level of 45,X was 14% that could be observed in the weighted Log2 ratio plot and the BAF plot of chromosome X, and SNP-array additionally detected a LB CNV on chromosome 16 (arr[hg19] 16q23.1(77,162,977–79,080,183)x3,1.917 Mb, maternal origin); (B) For the case of 47,XY, + 20[6]/46,XY[44], the mosaic level of 47,XY, + 20 was 12% that could not be observed in the weighted Log2 ratio plot and the BAF plot of chromosome 20, and SNP-array additionally detected two large segments of LOH on chromosome 20 (arr[hg19] 20q11.21q13.2(29,510,306–52,406,127) hmz, 22.895Mb, maternal origin;arr[hg19] 20p12.1p11.1(16,873,264–26,266,313) hmz, 9.393 Mb, maternal origin); (C) For the case of 47,XX, + 18[2]/46,XX[68]; the mosaic level of 47,XX, + 18 was 2.9% that could not be observed in the weighted Log2 ratio plot and the BAF plot of chromosome 18, and the SNP-array result is normal; (D) For the case of 47,XX, + 2[4]/46,XX[55], the mosaic level of 47,XX, + 2 was 6.8% that could not be observed in the weighted Log2 ratio plot, and the BAF plot of chromosome 2 could give some hint on mosaicism, while the SNP-array result is normal for mosaicism level less than 30% was not reported. [file Image_1.JPEG]
