# Supplementary material for: Clinical Utility of SNP Array Analysis in Prenatal Diagnosis: A Cohort Study of 5000 Pregnancies
Source: Front Genet. 2020 Nov 6;11:571219. doi: 10.3389/fgene.2020.571219 (PMC7677511; doi:10.3389/fgene.2020.571219)
Supplement: Supplementary file 2 [file Table_1.docx]

**Supplementary table 1. SNP-array confirmed CNVs in 47 cases with CNVs or trisomy detected by NIPT**

| **Case** | **NIPT results^a^** | **SNP-array results** | **Interpretation^b^** | **Karyotyping results** |
| --- | --- | --- | --- | --- |
| 1 | gain(chr22:20M-22M, 2M) | arr[hg19] 22q11.21(18,648,855-21,800,471)x3,3.152Mb； arr[hg19] 19q13.43(57,466,252-58,706,807)x3,1.241Mb | P | 46,XX |
| 2 | loss(chr18:67M-71M, 4M) | arr[hg19] 18q22.1(62,532,142-66,167,731)x1,3.636Mb | VOUS | 46,XY |
| 3 | loss(9p21.1)(28.5M-32M, 3.5M) | arr[hg19] 9p21.1(28,742,800-29,780,373)x1,1.038 Mb； arr[hg19] 9p21.1(30,547,485-31,996,569)x1,1.449 Mb | VOUS | 46,XX |
| 4 | Trisomy 21: Z=3.46 | arr[hg19] 21q21.1q21.2(21,232,613-25,143,119)x3,3.911 Mb | VOUS | 46,XY |
| 5 | gain(14q22.1-q24.1, 17.79M) | arr[hg19]14q21.3q24.1(50,655,302-68,867,256) x 3,18.212Mb | LP | 46,XX,dup(14)(q21.3q24.1) |
| 6 | gain(2q35-q36.3, 11.95M) | arr[hg19] 2q35q36.3(217,937,052-227,121,918)x3,9.185Mb | VOUS  (de novo) | 46,XY,dup(2)(q35q36.3) |
| 7 | gain(14q24.3-q32.12,15.30M); gain(14q32.12-q32.33,13.64M) | arr[hg19] 14q24.3q32.2(78,704,070-100,546,591)x3,21.843 Mb | LP(de novo) | 46,XY,der(14)add(14)(q32) |
| 8 | gain(16p13.12-p12.3, 5.51M) | arr[hg19] 16p13.11p12.3(15,481,747-18,242,713)x3,2.761Mb | LP(maternal origin) | 46,XX |
| 9 | gain(18p11.32-p11.22, 6.17M) | arr[hg19] 18p11.31(3,057,038-3,706,905)x3,650 Kb | LB | 46,XY |
| 10 | Trisomy 18 ^c^ | arr[hg19] 18q11.1q23(18,534,783-78,013,728)x3,59.479Mb；arr[hg19] 18p11.32 p11.21(136,304-15,079,294)x1,14.943 Mb | P | 46,XX,i(18)(q10) |
| 11 | gain(12q21.2-q21.31)(78.5M-84.5M,6M) | arr[hg19] 12q21.2q21.31(78,770,625-84,470,319)x3,5.700 Mb | VOUS | 46,XX |
| 12 | loss(12q13.3-q14.1)(57.5M-61.5M, 4M) | arr[hg19] 12q14.1(58,458,371-61,582,295)x1,3.124 Mb | VOUS | 46,XY |
| 13 | gain(7q31.31)(117.5M-119.5M, 2M) | arr[hg19] 7q31.31(117,614,219-118,512,894)x3,899 Kb | LB | 46,XY |
| 14 | Trisomy 18 ^c^ | arr[hg19] 18q22.3q23(69,997,897-74,381,199)x3,4.383Mb | VOUS | / ^d^ |
| 15 | gain(16p13.11-p12.3)(15.5M-18.5M, 3M) | arr[hg19] 16p13.11p12.3(15,319,277-18,242,713)x3,2.923Mb | LP | 46,XY |
| 16 | loss(8p23.3-p23.1,10.25M) | arr[hg19] 8p23.3p23.1(158,048-9,974,020)x1,9.816Mb | LP(de novo) | 46,XX,de1(8)(p23) |
| 17 | gain(2p12)(78M-80M, 2M) | arr[hg19] 2p12(78,631,709-79,973,436)x3,1.342 Mb | LB(maternal origin) | 46,XY |
| 18 | gain(chr12:85M-90M, 5M) | arr[hg19] 12q21.31(83,354,335-85,287,215)x3,1.933 Mb | LB(maternal origin) | 46,XY |
| 19 | loss(chr11:132.3M-133.5M, 1.2M) | arr[hg19] 11q25(132,336,926-133,495,443)x1,1.159 Mb | VOUS | 46,XX |
| 20 | gain(18q12.3, 2.17M) | arr[hg19] 18q12.3(40,379,514-41,410,107)x3,1.031Mb | LB(maternal origin) | 46,XY |
| 21 | Trisomy 11: Z=6.09 | arr[hg19] 11p11.12(49,193,984-51,238,712)x3,2.045 Mb | VOUS | 46,XX |
| 22 | gain(chr2:10M-11.9M, 1.9M) | arr[hg19] 2p25.1(10,076,854-11,861,320)x3,1.784 Mb | LB(maternal origin) | 46,XX |
| 23 | ChrX+(Y):Z=3.35 | arr[hg19] Xq21.2q21.31(85,393,530-89,136,096)x2,3.743Mb；arr[hg19] Xq26.3q27.1(137,899,736-139,867,922)x2,1.968Mb；arr[hg19] Xq27.3(142,856,383-144,729,248)x2,1.873Mb | LB(maternal origin) | 46,XY |
| 24 | gain(chr18:34.5M-35.5M,1M) | arr[hg19] 18q12.2(33,693,479-35,032,138)x3,1.339Mb | VOUS | 46,XY |
| 25 | gain(2p25.3-p25.2)(3M-5M, 2M) | arr[hg19] 2p25.3(3,068,861-4,074,083)x3,1.005Mb | LB(maternal origin) | 46,XY |
| 26 | gain(2q12.2-q12.3)(106.5M-108.5M, 2M) | arr[hg19] 2q12.2q12.3(106,873,992-108,527,327)x3,1.653Mb | VOUS | 46,XY |
| 27 | gain(21q21.1)(21M-23M, 2M) | arr[hg19] 21q21.1(20,993,165-22,638,334)x3,1.645Mb | LB(maternal origin) | 46,XX |
| 28 | gain(chr2:196.9M-205.2M, 8.3M) | arr[hg19] 2q33.2q33.3(203,556,717-205,385,454)x3,1.829Mb | VOUS | 46,XY |
| 29 | gain(20p12.2-p12.1)(11M-13.5M, 2.5M) | arr[hg19] 20p12.2p12.1(11,126,968-13,224,651)x3,2.098 Mb | VOUS | 46,XX |
| 30 | loss(18p11.32-p11.21,14.45M) | arr[hg19] 18p11.32q11.1(136,227-14,945,294)x1,14.809Mb；arr[hg19] 9q34.3(140,064,478-141,018,648)x3,954Kb | P, VOUS | 46,XY,del(18)(p11.3q11.1) |
| 31 | ChrX-: Z=-16.8 | arr[hg19] Xq26.2q28(131,287,664-155,233,098)x1,23.945Mb | P(maternal origin) | 46,X,del(X)(q26.2) |
| 32 | loss(10q26.13-q26.3)(125M-135.5M, 10.5M) | arr[hg19] 10q26.13q26.3(124,556,198-135,426,386)x1,10.870 Mb | P | / ^d^ |
| 33 | gain(7p21.3)(7.5M-9.5M, 2M) | arr[hg19] 7p21.3(7,902,686-8,584,911)x3,682Kb | VOUS | 46,XX |
| 34 | ChrX-: Z=-3.89 | arr[hg19] Xq27.1(138,536,878-139,230,825)x3,694Kb | LB(maternal origin) | 46,XX |
| 35 | loss(13q33.1-q33.2)(103M-107M, 4M) | arr[hg19] 13q33.1q33.2(103,476,903-106,713,312)x1,3.236Mb | LB(maternal origin) | 46,XY |
| 36 | gain(chr16:1M-4M,3M) | arr[hg19] 16p13.3(1,817,430-2,663,367)x3,846Kb | LB(maternal origin) | 46,XX |
| 37 | gain(11q23.3-q25)(119.5M-135M,15.5M) | arr[hg19] 11q23.3q25(116,683,754-134,937,416)x3,18.254 Mb; arr[hg19] 7q36.1q36.3(151,093,310-159,119,707)x1,8.026 Mb | P(de novo) | 46,XX,der(7)t(7;11)(q36;q23) |
| 38 | gain(chr15:53M-55M, 2M) | arr[hg19] 15q21.3(54,167,340-54,923,946)x4,757 Kb | LB(maternal origin) | / ^d^ |
| 39 | gain(chr10:2.34M-3.62M, 1.28M) | arr[hg19] 10p15.3p15.2(2,348,986-3,555,821)x3,1.207 Mb | VOUS | 46,XY |
| 40 | loss(chr13:47.6M-48.6M, 10M) | arr[hg19] 13q14.2(47,710,296-48,603,772)x1,893Kb | VOUS | 46,XY |
| 41 | ChrX-: Z=-5.12 | arr[hg19] Xp22.33p21.1(168,551-37,151,011)x1,6.982Mb； arr[hg19] Xp21.1q28(37,163,444-155,233,098)x3,118.069 Mb | P(de novo) | 46,X,idic(X)(p21.1) |
| 42 | gain(15q26.2-q26.3)(96.5M-99M, 2.5M) | arr[hg19] 15q26.2(96,465,787-97,279,457)x3,814Kb | VOUS | 46,XY |
| 43 | gain(1q23.3)(161M-163.5M, 2.5M) | arr[hg19] 1q23.3(161,676,575-162,260,959)x3,584Kb | VOUS | 46,XY |
| 44 | gain(chr22:20.3M-21.8M, 1.5M) | arr[hg19] 22q11.21(20,716,902-21,459,713)x3,742 Kb | VOUS | 46,XX |
| 45 | loss(13q21.33)(70M-72M, 2M) | arr[hg19] 13q21.33(70,438,851-71,704,967)x1,1.266Mb | VOUS | 46,XX |
| 46 | gain(12p13.31-p12.3,8.29M); gain(5p15.33-p14.2,24.17M) | arr[hg19] 12p13.31p13.2(7,900,183-12,751,609)x3,4.851Mb | VOUS | 46,XY,dup(12)(p13.2p13.31) |
| 47 | gain(15q11.2-q13.3,8.62M) | arr[hg19] 15q11.2q13.3(22,770,421-32,444,043)x4,9.674Mb | P | 47,XX,+mar |

1. The location and range of CNVs detected by NIPT using a low-coverage whole-genome sequencing method could only be estimated roughly.
2. CNVs were classified into four groups according to interpretation: P, pathogenic; LP, likely pathogenic; VOUS, variation of uncertain significance; LB, likely benign. And the origin of CNVs were indicated if parental samples were further analyzed by SNP-array.
3. The NIPT results of cases 10 and 14 were trisomy 18 performed in other center, and the Z-score were not recorded.
4. G-banded karyotyping were not conducted for cases 14, 32, and 38.
